# Prediction of the toughness of date palm fruit

**DOI:** 10.1038/s41598-024-81881-2

**Published:** 2025-01-09

**Authors:** Ahmed M. Hassan, Mohamed M. Ibrahim, Mohamed Ghonimy, Maher Fathy

**Affiliations:** 1 Department of Agricultural Engineering, Faculty of Agriculture, Cairo University, Giza, Egypt; 2 Department of Agricultural and Biosystems Engineering, College of Agriculture and Food, Qassim University, P.O. Box 6622, Buraydah, 51452 Saudi Arabia; 3 Department of Water Relations and Field Irrigation, Agricultural and Biological Research institute, National Research Centre, Giza, Egypt

**Keywords:** Date palm, Fruit, Physical and mechanical, Modeling, Toughness, Mechanical engineering, Rheology

## Abstract

This study aimed to predict the toughness of date palm fruit (Barhi, Saqie, and Khodry varieties) at different ripening stages (Khalal, Rutab, and Tamar) using Hertz Theory by evaluating the physical and mechanical characteristics of the fruits. Physical measurements revealed that high moisture content in the Khalal stage led to larger dimensions and mass across all varieties, with Barhi dates showing a moisture content of 63.31%, which decreased to 32.60% at Rutab as the fruit dehydrated. This moisture reduction also impacted other characteristics, such as bulk density, volume, and flesh thickness, resulting in a concentrated, denser structure in the Tamar stage. Mechanical analysis demonstrated significant softening in all varieties as they transitioned from Khalal to Rutab stages, with Barhi dates’ modulus of elasticity dropping from 548.83 to 16.72 kPa. Similarly, bioyield and rupture stress decreased, highlighting the influence of moisture loss on textural characteristics. Saqie and Khodry varieties followed similar trends, with initial softening from Khalal to Rutab, and a slight firmness increase at Tamar due to dehydration. The force-deformation curves and toughness measurements confirmed these findings, showing significant reductions in toughness from Khalal to Rutab, with a slight increase in Tamar. These results underscore the pronounced textural and structural changes as dates ripen, influencing their suitability for different applications. Also, the measured toughness (*τm*) closely aligned with predicted toughness (*τp*), with significant textural differences observed across varieties and ripening stages. For all varieties, the τp is approximately 0.00–9.24% lower than the *τm*. Finally, the derived equation can be used with enough confidence to theoretically predict the toughness of date palm fruit.

## Introduction

Date palm is considered very important crops in agricultural and economic activities in many regions, particularly within the Arab and Islamic world. Egypt is high rank in term of date production that amounts to more 1.7 million tons almost 21% of the world production^1^. In Kingdom of Saudi Arabia, the date palm cultivated area is approximately 156,460 hectares with a total annual production of 1.61 million tons^2^. The focus on date palm cultivation and date production in Saudi Arabia due to its religious, social, and economic values, so it is part of the Kingdom’s Vision 2030, which aims to diversify national income sources and develop the agricultural sector, thereby the total quantity of exports date reached over 321,000 tons, contributing to a revenue of 324.6 million USD^3^. Among the numerous varieties, Barhi, Saqie, and Khodry stand out due to their unique qualities and widespread popularity in Saudi Arab. Each of these varieties undergoes significant transformations across three primary ripening stages: Khalal (mature but unripe), Rutab (ripe and soft), and Tamar (full mature stage). Understanding the physical, and mechanical characteristics of these date varieties at each ripening stage are crucial for optimizing their handling, processing, and different utilization. Recent studies underscore the importance of these characteristics in enhancing agricultural practices and processing technologies. Dimensions are important when deciding on a machine’s aperture size, especially when it comes to material separation, as Solomon et al.^4^ highlighted. When developing machine components, these dimensions might be utilized. Świąder et al.^5^ have emphasized the considerable variability in physical characteristics among different date cultivars. These include size, shape, color, texture, and moisture content, which significantly impact consumer preferences, storage, transportation, and processing methods. Chaudhari and Waghmare^6^ have focused on developing methodologies for quality assessment and grading of dates based on their physical attributes. Understanding these characteristics enables producers and exporters to classify dates according to their market suitability, ensuring consumer satisfaction and market competitiveness. Forsido et al.^7^ and Greiby and Fennir^8^ have investigated the effects of temperature, humidity, and packaging materials on the physical attributes and storage stability of dates. This knowledge is essential for optimizing storage conditions and prolonging the shelf life of dates to maintain the date quality, thereby minimizing post-harvest losses. Thomas et al.^9^ has explored various processing methods, such as drying, extrusion, and fermentation, to enhance the nutritional value, sensory characteristics, and marketability of date-based products. These studies highlight the importance of tailoring processing parameters to the specific physical characteristics of dates to achieve desired product outcomes. Also, mechanical characteristics are equally critical and important issue. Alhamdan et al.^10^ analyzed the texture profile parameters—brittleness, hardness, cohesiveness, elasticity, and adhesiveness—of eight Saudi date cultivars (Bari, Khudari, Khlass, Serri, Sukkari, Suffri, Saqie, and NubotSaif) at three maturity stages: Khalal, Rutab, and Tamer. The study found that five cultivars had higher firmness at the Tamer stage compared to the Rutab stage, while three cultivars showed lower firmness at Tamer than at Rutab. Significant differences in firmness were noted at the Khalal stage for all cultivars relative to the other stages. All cultivars, except Suffri, exhibited greater cohesiveness at Tamer than at the earlier stages. Overall, notable differences in firmness, adhesiveness, resilience, chewiness, and gumminess were observed at the Khalal stage compared to both Rutab and Tamer stages. Wang et al.^11^ investigated the compressive strength and deformation characteristics of Barhi, Saqie, and Khodry dates, concluding that a thorough understanding of these mechanical characteristics is essential for improving transportation and storage methods. Ensuring that dates reach consumers in the best possible condition requires precise knowledge of their mechanical behavior at different ripening stages. Elastic characteristics, which describe how dates respond to stress and strain, it is play a significant role in mechanical processing such as pitting, pressing, and molding. Ibrahim and Hassan^12^ and Khodabakhshian and Khojastehpour^13^ explored the elastic characteristics of dates, discovering significant variations between the Rutab and Tamar stages. These variations affect how dates can be pressed and molded into different products, providing valuable insights for optimizing the production of date-based items like paste and syrup. Further, Ghonimy and Kassem^14^ focused on the elastic modulus of different date varieties, demonstrating that understanding the elastic behavior of dates is crucial for designing equipment and processes that minimize waste and improve product consistency during mechanical processing. Additionally, they stated that there are notable variations in fruit mass, flesh mass, volume, moisture content, fruit dimensions, thickness of the flesh, projected area of the fruit, and fruit elasticity among the various date fruit producing zones. Toughness and resilience are both characteristics that describe how a material responds to external forces, but they represent different aspects of a material’s behavior. Toughness is a measure of the ability of a material to absorb energy and plastically deform without fracturing. It is the amount of energy per unit volume that a material can absorb before rupturing. Toughness is typically measured by the area under the stress-strain curve of a material. Materials that can undergo large deformations without breaking tend to have high toughness. Toughness is important in applications where materials are subjected to impact or high loads^15^. Moreover, the prediction of the toughness of date palm fruit according to Hertz theory has gained significant attention. Hertz theory, traditionally applied to understand contact mechanics, provides a framework for predicting the toughness and mechanical behavior of dates under compression. A study by Fonsso et al.^16^ successfully applied Hertz theory to model the deformation and toughness of date fruits, offering a predictive tool that can enhance quality control and process optimization. This theoretical approach, combined with empirical data, can lead to more efficient handling and processing protocols, minimizing damage and improving the overall quality of the dates. Thus, the main objective of this study is to predict the toughness of date palm fruit (Barhi, Saqie, and Khodry varieties) at different ripening stages (Khalaal, Rutab, and Tamar) according to Hertz Theory by measuring and analyzing the physical and mechanical characteristics of date palm fruits.

## Materials and methods

This study was conducted on three date palm fruit varieties (Barhi, Saqie, and Khodry) at three ripening stages (Khalal, Rutab, and Tamar). Each variety was analyzed to observe the physical and mechanical changes that occur as the fruits progress through these stages of ripeness. However, the Barhi variety lacks the ability to dry out sufficiently to reach the Tamar stage, which is characterized by a significant reduction in moisture content and a chewy texture. Consequently, studying the Tamar stage for Barhi dates is not applicable, as this variety is predominantly harvested and enjoyed before it reaches such an advanced stage of ripeness.

Determining the ripening stages of date fruits involves assessing various physical, chemical, and sensory characteristics. Key methods for classification include observing morphological traits, such as changes in skin color from green (immature) to yellow, amber, or brown (ripe) as well as measuring size and shape alterations during maturation. In this study, dates were classified into specific stages: Khalal dates appear yellow or light brown, remaining firm but beginning to ripen; Rutab dates are fully soft, mature, and often dark brown or black; and Tamar dates are fully dried, characterized by concentrated sugars^13^.

This investigation was divided into three stages. The first stage was determined the physical characteristics of the date palm fruits. The second stage was estimated the mechanical characteristics of the date fruits. While the third stage was predicted the toughness of date palm fruit according to Hertz Theory.

For different varieties, one hundred date fruits were gifted from a private farm (Sheikh Mohammed Abdul Aziz Al-Rajhi), Qassim region at different ripening stage. These fruits were gathered from different locations to ensure a representative sample. The fruits were then air-transported in the same day to the Agricultural Engineering department, Faculty of Agriculture, Cairo University for further analysis and study. This meticulous handling and transportation process ensured that the dates maintained their integrity and freshness for accurate evaluation in the study.

### The first step: the physical characteristics determination of date fruits

**Fig. 1:**
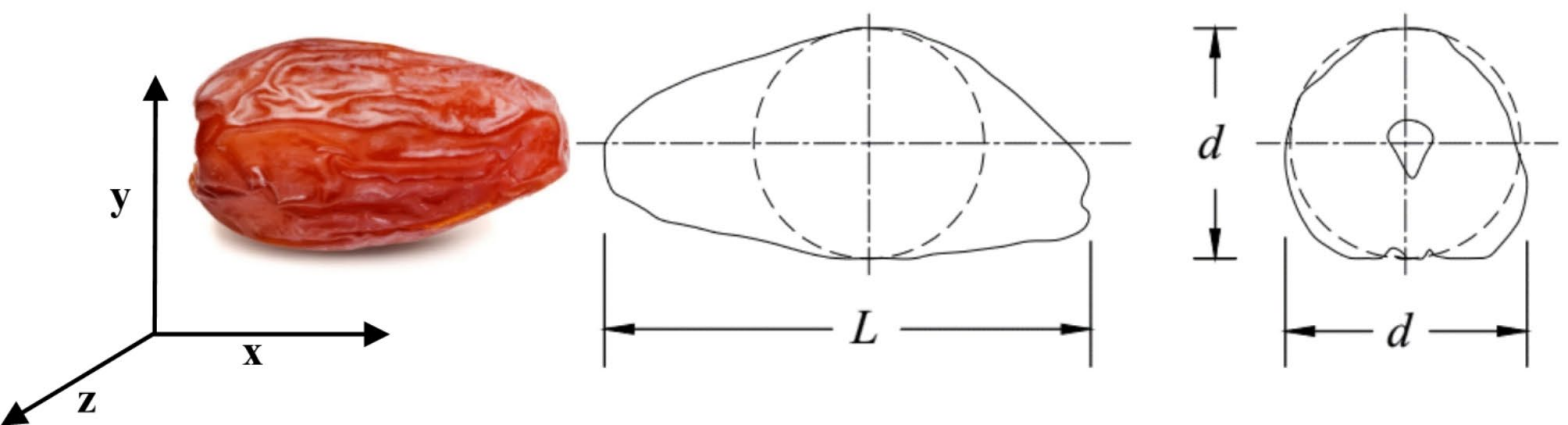
The dimensions of the date fruit, where *x*-axis is fruit length (*L*), *y*-axis and *z*-axis are fruit diameter.

Date fruit dimensions (length and diameter) were measured with a Vernier caliper to an accuracy of 0.1 mm. Figure 1 shows the three dimensions of date fruit. The moisture content of the date flesh was determined following AOAC procedures^17^, which involved drying the samples at 70 °C for 48 h. The date fruit mass was measured using a digital balance accurate to 0.01 g, and the bulk density was calculated using the mass and volume of the date fruit according to the formula *ρ*_*b*_ = *m/V*, where *ρ*_*b*_ is the bulk density in g cm^–3^, *m* is the mass in grams, and *V* is the volume in cm^3^.

### The second step: the mechanical characteristics of date fruits

The mechanical characteristics of date fruits encompass both compression tests and the elastic characteristics of the fruit. The compression tests involve using a universal testing machine to measure how the fruit responds to uniaxial compression load, providing valuable data on its strength and deformation characteristics. Also, the elastic characteristics of date fruit are assessed through stress-strain tests, which measure the fruit’s ability to return to its original shape after being deformed. These combined tests offer a comprehensive understanding of the mechanical behavior of date fruit at various stages of date fruit ripening.

**Fig. 2:**
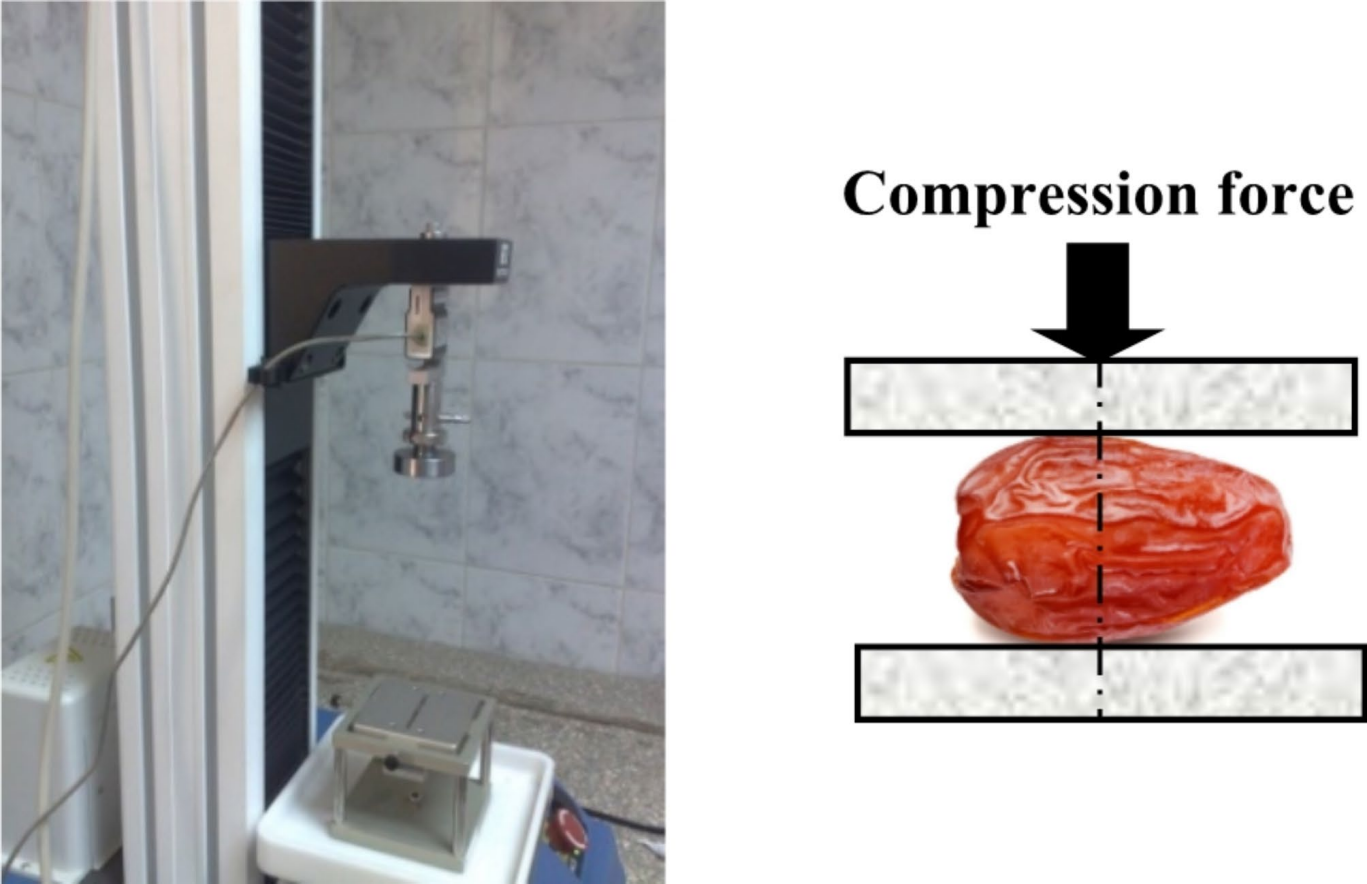
Date fruit loaded between the two parallel plates by using universal testing machine.

For compression test, a universal testing machine (Instron-1000 N) was used to perform the parallel plate compressive test in order to ascertain the mechanical characteristics. Date fruits were squeezed uniaxially to a total deformation of 10 mm at a cross-head speed of 0.5 mm • s^–1^. A date flesh slab that was put on a mounted, fixed table was compressed by a plate with a 7.5 cm diameter, loading the contact surfaces parallel to the compression surfaces as shown in Fig. 2. At room temperature (23 °C), random samples of ten fruits from each variety at each ripening stage were utilized for the compression testing. By covering the plunger disk surface with white paper, gently pressing the horizontally oriented upper longitudinal fruit surface into an ink stamp, and then allowing the plunger to come into contact with the fruit surface, the contact area between each tested fruit surface and the parallel-plate disk surface was experimentally determined. Once the resulting contact area has been traced on the white paper, specialized software was used to properly estimate the scanned surface area.

**Fig. 3:**
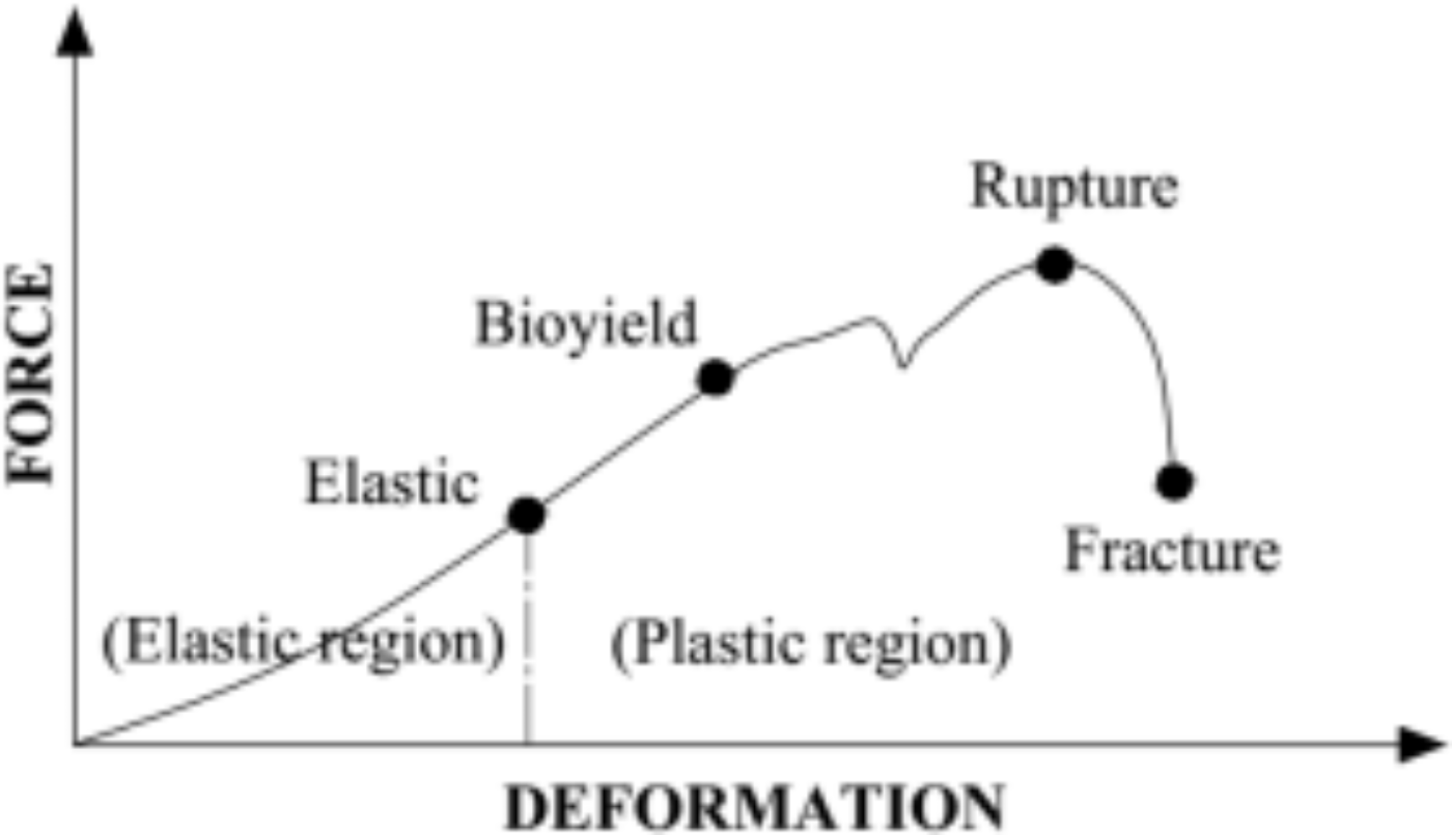
A force deformation curve of agricultural materials^15^.

Figure 3 depicts a typical force deformation curve^15^ for the mechanical characteristics of date fruits. The force deformation curve had two peak locations, as can be shown. The yield point at which fruit damage first appeared is represented by the first peak. With respect to the greatest compressive force, the second peak corresponds.

The mechanical characteristics that were determined for the stress-strain tests were the rupture energy toughness (*RE*), firmness coefficient (*fc*), rupture stress (*σr*), rupture strain (*εr*), bioyield stress (*σb*) bioyield strain (*εb*), and modulus of elasticity (*E*). The elasticity modulus is a reliable measure of the elasticity of materials, described by Hooke’s law and modeled as a spring without a damper. For compressive stress, Young’s modulus (*E*) is given by the equation *E = σ/ε*, where *E* is Young’s modulus in kPa, *σ* is compressive stress in kPa, and $\epsilon$ is strain in mm • mm^–1^. Strain was calculated by dividing the deformation of the fruit by its initial average thickness, with deformation (*Δl*) measured in mm and original thickness (*l*) also in mm. The stress values were determined by dividing the force on one fruit by its projected area. The coefficient of firmness (*fc*) was calculated as the average slope of the force-deformation curve from zero to the rupture point^18^.

### Third step: predicting the toughness of date palm fruit according to Hertz theory

Predicting the toughness of date palm fruit based on Hertz Theory is essential for improving post-harvest handling, optimizing packaging, and enhancing processing techniques in the food industry.

#### Mathematical analysis approach

The toughness of a material can be defined as the total energy absorbed per unit volume until the rupture point. This can be expressed as the area under the force-deformation curve up to the point of rupture. Mathematically, toughness (*τ*) can be represented from Eq. (1):

1 $$\tau =\mathop \smallint \limits_{0}^{{{D_r}}} {F_r}dx$$

where *F*_*r*_ is the rupture force, N; and *D*_*r*_ is the deformation at the rupture point, m.

Using Hertz’s theory, we can relate the force to deformation in an elastic contact scenario and use these relations to calculate the toughness.

#### General assumptions and simplifications

Some assumptions and simplifications were made in order to facilitate the mathematical manipulation as follows:

1. The date fruit is homogeneous and isotropic.
2. The material behaves elastically up to the point of rupture.
3. The contact surfaces are smooth and frictionless.
4. The deformations are small compared to the dimensions of the fruit.
5. The rupture force (*F*_*r*_) and deformation at rupture (*D*_*r*_) are known.

#### Mathematical analysis steps

1. Young’s modulus (*E*).

In the elastic region, the relationship between stress and strain is line *σ = E*$\epsilon$, the lateral strain ($\epsilon$) can be related to the change in diameter$~\epsilon =\frac{{d - {d_0}}}{{{d_0}}}$. while the stress (*σ*) is related to the applied force and contact area and equal to $\sigma =\frac{F}{A}$. Thus,

2 $$E=\frac{\sigma }{\epsilon }$$

2. Effective elastic modulus (E^*^).

Equation (3) is used to compute the effective elastic modulus (*E*^∗^), which takes into consideration the combined effects of the material’s elasticity and Poisson’s ratio (*v*).

3 $${E^*}=\frac{E}{{1 - {v^2}}}$$

This parameter is crucial for understanding the material’s behavior under stress and for simplifying the subsequent calculations.

3. Applied force (*F*) and deformation (*δ*).

In this step, derive the relationship between the applied force and the resulting deformation using Hertz’s theory. The theory provides the deformation *δ* as:

4 $$\delta ={\left( {\frac{{9{F^2}}}{{16~{E^{*2}}R}}} \right)^{1/3}}$$

Raise both sides to the power of 3 to get rid of the cube root:

$${\delta ^3}=\frac{{9{F^2}}}{{16~{E^{*2}}R}}$$

Rearrange the equation to solve for *F* gives:

5 $$F={\left( {\frac{{16~{E^{*2}}R{\delta ^3}}}{9}} \right)^{1/2}}$$

Equation (5) correlates the contact force to the deformation, helping us understand how the fruit will react to the applied stress.

4. Determine the toughness.

To determine the toughness, substituting from Eq. (5) into Eq. (1) gives:

$$\tau =\mathop \smallint \limits_{0}^{{{D_r}}} {\left( {\frac{{16~{E^{*2}}R{\delta ^3}}}{9}} \right)^{1/2}}d\delta$$

$$\tau ={\left( {\frac{{16~{E^{*2}}R}}{9}} \right)^{1/2}}\mathop \smallint \limits_{0}^{{{D_r}}} {\delta ^{3/2}}d\delta$$

Solving this integral gives:

6 $$\tau =\frac{2}{5}{\left( {\frac{{16~{E^{*2}}R}}{9}} \right)^{1/2}}{D_r}^{{5/2}}$$

Substituting from Eq. (3) into Eq. (6) gives,

$$\tau =\left( {\frac{8}{{15}}} \right)\left( {\frac{{E{R^{1/2}}}}{{1 - {v^2}}}} \right){D_r}^{{5/2}}$$

To express *τ* in J • m^–3^, we divide by the fruit volume *V*:

Thus,

7 $$\tau =\left( {\frac{1}{{1000}}} \right)\left\{ {\left( {\frac{8}{{15}}} \right)\left( {\frac{{E{R^{1/2}}}}{{1 - {v^2}}}} \right){D_r}^{{5/2}}} \right\}/V$$

Where τ is the date palm fruit toughness, kJ • m^–3^; *E* is the Young’s modulus of the date fruit, Pa; *R* is the radius of curvature of the contacting surface, m; ν is Poisson’s ratio, dimensionless; *D*_*r*_ is the deformation at the rupture point, m; and *V* is the date palm fruit volume, m^3^.

Toughness was determined in this work utilizing two techniques. First, measured toughness (*τm*) is used. It is most likely through direct mechanical testing that the measured toughness levels were achieved. By computing the area under the force-deformation curve, it was ascertained. Conversely, the projected toughness (*τp*) is the second technique. Equation (7), which is based on the qualities of the date palm fruit, was utilized to compute it by theoretical models or empirical formula.

### Statistical analysis

Using the MStat-C statistical program, a randomized complete block design was used for the statistical analysis. LSD and Duncan’s multiple range comparison tests were used to find means that differed at a 5% probability level or less^19^.

## Results and discussion

### Physical characteristics of date fruits

The average values of date fruit length, width, thickness, mass, flesh mass, flesh thickness, seed mass, and moisture content, are shown in Table 1. For Barhi variety, the physical characteristics of Barhi date at different ripening stages reveal significant changes. At the Khalal stage, Barhi dates are characterized by a high moisture content of 63.31%, indicating their early ripening phase. The high moisture content contributes to larger dimensions and mass, with an average length of 26.12 mm, diameter of 19.58 mm, and mass of 6.00 g. As the dates progress to the Rutab stage, there is a noticeable reduction in these characteristics: the length decreases to 23.71 mm, the diameter to 17.82 mm, and the mass to 5.60 g.

The volume and bulk density also decrease, reflecting the natural dehydration process and shrinkage associated with ripening. The significant drop in moisture content to 32.6% at the Rutab stage indicates a loss of water and a transition to a more concentrated form. These changes are statistically significant (*p* < 0.05), highlighting the substantial physical transformation from Khalal to Rutab. The decreasing flesh thickness and mass further support the notion of moisture loss and textural changes as the fruit matures. The slight increase in seed mass at the Rutab stage may be attributed to the relative concentration effect as the flesh dehydrates. The same trend was found with Saqie and Khodry varieties.

The results indicated that the physical characteristics of date palm fruits are significantly influenced by the ripening stage, with notable differences among the Barhi, Saqie, and Khodry varieties. The high moisture content in the Khalal stage contributes to larger dimensions and mass, which decreases as the fruit ripens and loses water. The Rutab stage is marked by a peak in moisture content and dimensions, indicating optimal ripeness. The Tamar stage, characterized by reduced moisture content and dimensions, represents the final ripening phase with a drier and more concentrated fruit. The significant differences observed in the physical characteristics across the stages (*p* < 0.05) highlight the impact of ripening on fruit characteristics. The high moisture content and flesh mass in the Rutab stage suggest that this is the ideal stage for consumption due to its juiciness and flavor. In contrast, the Tamar stage, with its lower moisture content and higher concentration of sugars, is more suitable for long-term storage and processing into date products.

**Table 1:** Physical characteristics of date palm fruit.

Characteristics	Barhi		Saqie			Khodry		
	Khalal	Rutab	Khalal	Rutab	Tamar	Khalal	Rutab	Tamar
*L*, mm	26.12 ± 1.98	23.71 ± 1.80	40.13 ± 0.41	37.90 ± 0.92	37.44 ± 0.61	42.04 ± 1.68	38.41 ± 1.14	38.24 ± 1.72
*d*, mm	19.58 ± 1.23	17.82 ± 1.11	20.31 ± 0.82	19.19 ± 0.68	18.88 ± 0.51	20.31 ± 0.80	19.31 ± 0.71	18.88 ± 0.71
*m*, g	6.00 ± 0.29	5.60 ± 0.22	10.33 ± 0.3	9.81 ± 0.21	9.81 ± 0.49	10.13 ± 0.80	9.19 ± 0.82	8.87 ± 0.91
*V*, cm^3^	5.32 ± 0.71	4.44 ± 0.60	8.71 ± 0.68	7.33 ± 0.59	6.90 ± 0.51	9.13 ± 0.81	7.50 ± 0.70	7.14 ± 0.51
*ρ*_*b*_, g • cm^–3^	0. 61 ± 0.09	0. 56 ± 0.08	0. 63 ± 0.10	0. 61 ± 0.09	0. 61 ± 0.08	0.59 ± 0.07	0.55 ± 0.09	0.54 ± 0.06
*T*_*f*_, mm	5.33 ± 0.09	4.87 ± 0.2	6.91 ± 0.42	6.20 ± 0.50	6.00 ± 0.41	7.40 ± 0.2	7.13 ± 0.08	7.04 ± 0.09
*m*_*f*_, g	4.89 ± 0.41	3.91 ± 0.41	9.09 ± 0.24	8.72 ± 0.31	8.80 ± 0.20	9.61 ± 1.04	8.51 ± 0.63	8.30 ± 0.90
*m*_*s*_, g	0.83 ± 0.05	1.02 ± 0.07	1.03 ± 0.04	1.02 ± 0.08	1.11 ± 0.07	0.72 ± 0.07	0.71 ± 0.32	0.64 ± 0.09
*MC*, %	63.31 ± 5.22	32.60 ± 8.21	62.40 ± 7.02	25.32 ± 6.81	24.78 ± 5.43	64.41 ± 6.51	29.10 ± 5.40	28.88 ± 6.50

*L*, fruit length; *d*, fruit diameter; *m*, fruit mass; *V*, fruit volume; *ρ*_*b*_, bulk density; *T*_*f*_, flesh thickness; *m*_*f*_, flesh mass; *m*_*s*_, seed mass; *MC*, moisture content.

Barhi exhibited the shortest fruit length at the Khalal stage (26.12 ± 1.98 mm) and Saqie the longest at the Tamar stage (42.04 ± 1.68 mm), with Khodry remaining relatively consistent across stages. These variations align with findings by Alrashidi et al.^20^, who reported that date varieties show significant differences in fruit dimensions, influenced by genetic and environmental factors. The fruit diameter showed a similar trend, with Saqie generally having the largest diameter, which is consistent with Ibrahim et al.^21^, who noted significant variation in date fruit sizes among different cultivars. The mass and volume measurements followed a pattern where Saqie exhibited higher values, particularly at the Tamar stage, supporting the observations of Hussain et al.^22^, who found that the ripening stage greatly affects the physical characteristics of dates. The bulk density values were relatively similar across varieties, slightly decreasing from Khalal to Tamar, which aligns with the findings of Alem et al.^23^. Flesh thickness and mass were highest in Saqie at the Tamar stage, correlating with the results of Hamad et al.^24^, who reported that later stages of maturity result in thicker and heavier flesh. Seed mass was slightly higher in Khodry and Saqie, consistent with the research by Ibrahim and Hassan^12^, who noted seed size variations among date varieties. Lastly, moisture content decreased significantly from Khalal to Tamar stages across all varieties, with Saqie having the highest moisture content at the Khalal stage, which supports the findings of Khodabakhshian and Khojastehpour^13^ on the moisture variation during the ripening process.

### Mechanical characteristics of date fruits

The mechanical characteristics of date fruits with different varieties at different ripening stages included elasticity modulus (*E*), coefficient of firmness (*fc*), bioyield stress (*σb*), rupture stress (*σr*), bioyield strain (*εb*), rupture strain (*εr*), Poisson’s ratio (*v*) and effective elastic modulus (E^*^) are shown in Table 2. It’s clear that there are significant differences in the mechanical characteristics of Barhi, Saqie, and Khodry dates across different ripening stages.

For Barhi variety, the mechanical characteristics of Barhi dates at the Khalal and Rutab stages exhibit significant changes, reflecting the textural transformations during ripening. At the Khalal stage, Barhi dates show a high modulus of elasticity (*E*) of 548.83 kPa, indicating a firm and rigid structure. This value decreases to 16.72 kPa at the Rutab stage, highlighting the softening of the fruit as it ripens. The firmness coefficient (*fc*) at the Khalal and Rutab stages are 11.34 and 0.77 N • mm^–1^ respectively, supporting the high firmness of Barhi dates at Khalal stage. The bioyield stress (*σb*) and bioyield strain (*εb*) also show significant differences, with *σb* dropping from 178.51 kPa at Khalal to 3.13 kPa at Rutab, and *εb* decreasing from 0.34 to 0.22 m • m^–1^. These changes indicate that Barhi dates become considerably softer and less resistant to deformation as they ripen. The rupture stress (*σr*) and rupture strain (*εr*) further corroborate this trend, with *σr* reducing from 203.83 to 5.52 kPa and *εr* from 0.44 to 0.25 m • m^–1^, respectively. The Poisson’s ratio (*v*) remains relatively constant, suggesting that the volumetric strain behavior does not change significantly during ripening. The effective elastic modulus (E*) follows the same trend as *E*, decreasing from 556.88 kPa at Khalal to 17.03 kPa at Rutab. The statistical analysis indicates that all these changes are significant (*p* < 0.05), reflecting the substantial softening and increased deformability of Barhi dates from Khalal to Rutab. These results highlight the impact of moisture loss and structural changes on the mechanical characteristics of Barhi dates during ripening. The same trend was found for Saqie and Khodry varieties.

**Table 2:** Mechanical characteristics of date palm fruit.

Characteristic	Barhi		Saqie			Khodry		
	Khalal	Rutab	Khalal	Rutab	Tamar	Khalal	Rutab	Tamar
*E*, kPa	548.83 ± 15.21	16.72 ± 1.54	832.90 ± 37.42	80.19 ± 1.90	134.68 ± 28.50	426.72 ± 23.71	30.61 ± 1.94	88.04 ± 2.02
*fc*, N • mm^–1^	11.34 ± 1.10	0.77 ± 0.11	17.56 ± 0.88	3.31 ± 0.21	3.59 ± 0.07	10.10 ± 0.43	1.79 ± 0.21	3.38 ± 0.21
*σb*, kPa	178.51 ± 13.50	3.13 ± 1.79	279.01 ± 18.37	23.23 ± 5.81	34.74 ± 12.10	140.60 ± 17.40	5.73 ± 2.92	22.42 ± 6.39
*εb*, m • m^–1^	0.34 ± 0.05	0.22 ± 0.01	0.38 ± 0.00	0.24 ± 0.01	0.32 ± 0.00	0.36 ± 0.00	0.19 ± 0.02	0.29 ± 0.02
*σr*, kPa	203.83 ± 11.10	5.52 ± 1.61	304.12 ± 14.00	34.18 ± 6.60	38.27 ± 9.31	166.20 ± 21.59	15.03 ± 3.20	33.91 ± 7.00
*εr*, m • m^–1^	0.44 ± 0.03	0.25 ± 0.00	0.44 ± 0.02	0.34 ± 0.02	0.36 ± 0.04	0.46 ± 0.00	0.35 ± 0.00	0.43 ± 0.01
*v*, dimensionless	0.12 ± 0.01	0.13 ± 0.00	0.11 ± 0.02	0.10 ± 0.00	0.14 ± 0.00	0.03 ± 0.02	0.04 ± 0.03	0.11 ± 0.02
E*,* kPa*	556.88 ± 18.40	17.03 ± 3.21	843.14 ± 22.60	81.00 ± 11.79	137.40 ± 6.74	427.04 ± 9.41	30.60 ± 10.03	89.11 ± 6.42

*E*, elasticity modulus; *fc*, coefficient of firmness; *σb*, bioyield stress; *εb*, bioyield strain; *σr*, rupture stress; *εr*, rupture strain; *σb*, bioyield stress; *εb*, bioyield strain; *ν*, Poisson’s ratio; E^*^, effective elastic modulus.

For Barhi dates, the substantial decrease in modulus of elasticity, bioyield stress, and rupture stress from the Khalal to Rutab stages underscores the softening and increased deformability due to moisture loss. The Saqie variety shows a non-linear pattern, with significant initial softening from Khalal to Rutab, followed by a slight increase in firmness at the Tamar stage, reflecting complex textural changes. Khodry dates exhibit similar trends, with initial softening followed by a firmer texture at the Tamar stage due to dehydration and concentration. The statistically significant differences (*p* < 0.05) across all measured characteristics highlight the impact of ripening on the mechanical characteristics of date fruits, which is crucial for optimizing harvesting, processing, and storage methods to maintain quality and shelf life.

The mechanical characteristics of the date palm fruits with different varieties (Barhi, Saqie, and Khodry) at different stages of maturity (Khalal, Rutab, and Tamar) reveal significant variations in elasticity modulus (*E*), coefficient of firmness (*fc*), bioyield stress (*σb)*, rupture stress (*σr*), bioyield strain (*εb*), rupture strain (*εr)*, Poisson’s ratio (*ν*), and effective elastic modulus (E*).Barhi showed a high modulus of elasticity at the Khalal stage (548.83 ± 15.21 kPa) and a significant reduction at the Rutab stage (16.72 ± 1.54 kPa), while Saqie exhibited the highest modulus at the Khalal stage (832.90 ± 37.42 kPa) and a notable decrease by the Tamar stage (134.86 ± 28.50 kPa), consistent with Hamad et al.^24^, who reported similar trends in modulus of elasticity across different stages of maturity. The bioyield stress values followed a similar pattern, with Saqie showing the highest stress at the Khalal stage (279.01 ± 18.37 kPa) and significantly lower values at the Tamar stage (34.74 ± 12.10 kPa), which aligns with the findings of Alem et al.^23^, indicating that ripening reduces the mechanical strength of dates. The rupture stress and strain values also reflect these trends, with Saqie showing higher rupture stress at the Khalal stage (304.12 ± 14.00 kPa) and decreasing at the Tamar stage (38.27 ± 9.31 kPa), supporting the observations of Khodabakhshian and Khojastehpour^13^ regarding the reduction in mechanical characteristics as dates ripen. Poisson’s ratio (*ν*) showed minimal variation among the varieties and stages, with the exception of Saqie at the Rutab stage, which had an unusually low value (0.03 ± 0.02), consistent with the variability^25^. The effective elastic modulus (E*) values followed the same decreasing trend from Khalal to Tamar stages across all varieties, further confirming the observations by Ibrahim et al.^21^ that the mechanical characteristics of dates fruits diminish as they ripen.

### The toughness of date palm fruit

#### Force-deformation curve

**Fig. 4:**
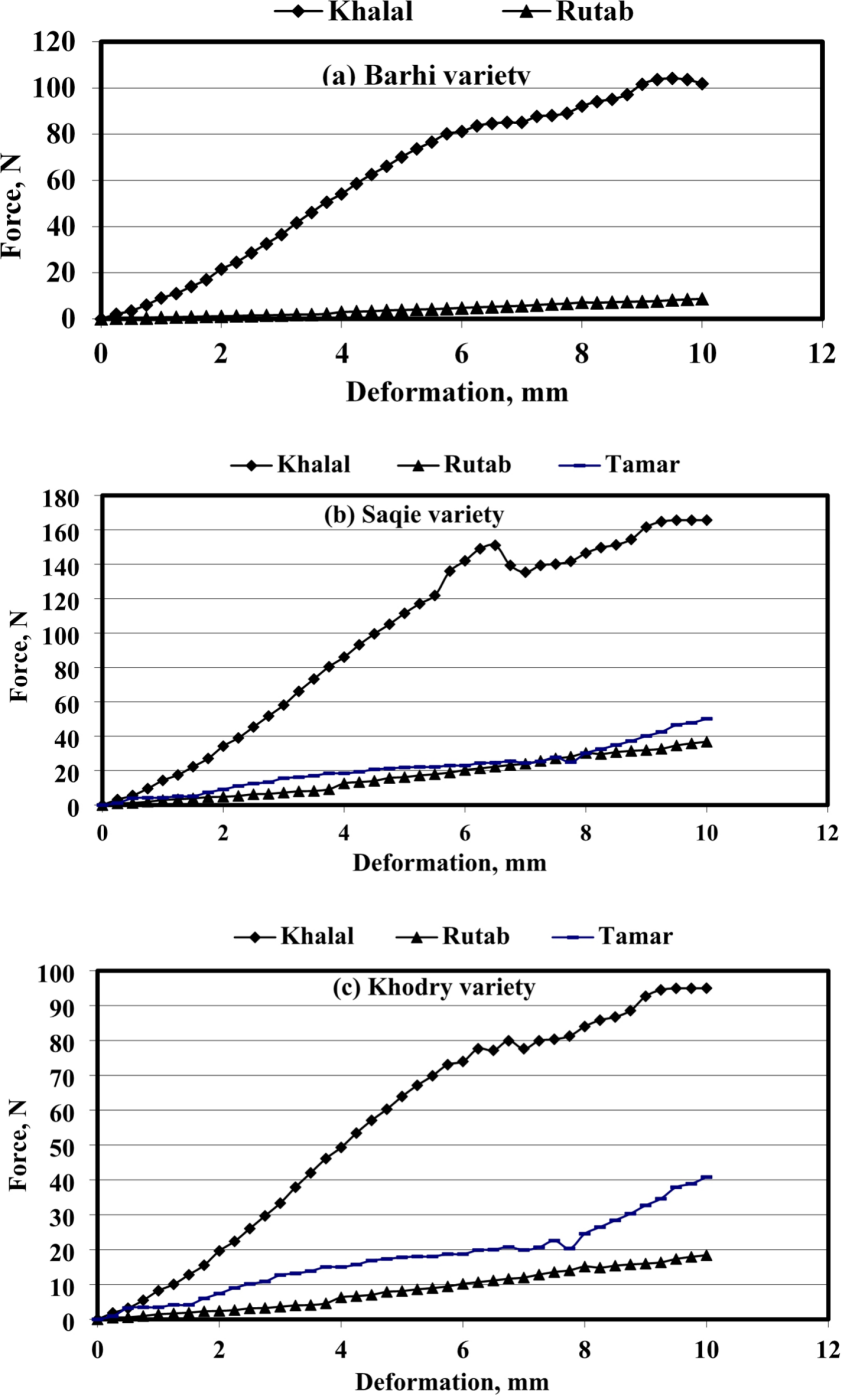
Force-deformation curves of date fruit varieties at different ripening stages.

Figure 4a–c show the Force-deformation curve of palm date fruit with different varieties (Barhi, Saqie, and Khodry) at three ripening stages. Evidently, prior to the so-called proportional limit (Pl). The elastic limit is, in general, the point at which the load may be removed without the date returning to its initial shape, or it is the highest force that can be created without causing a permanent setting when the load is completely removed. The statistical analysis (at 5% level) for the three kinds (Khalal, Rutab, and Tamar) revealed significant differences for most of the elasticity characteristics of date fruits during the three stages of ripening.

For Barhi variety, it is evident that both the force at bioyield and force at rupture decrease from the Khalal to Rutab stages. At the Khalal stage, the force at bioyield is 85.10 N and drops sharply to 2.17 N at the Rutab stage. Similarly, the force at rupture decreases from 97.14 N at Khalal to 3.83 N at Rutab. The deformation at bioyield and rupture also shows a notable reduction, indicating that the fruit becomes significantly softer and less resistant to deformation as it ripens. These changes are statistically significant (*p* < 0.05), underscoring the pronounced textural transformations due to the increased moisture content and breakdown of cell structure. For Saqie variety, the force at bioyield and rupture also exhibit significant reductions as the fruit ripens from Khalal to Tamar stages. The force at bioyield decreases from 141.70 N at Khalal to 15.77 N at Rutab and further to 22.99 N at Tamar. The force at rupture follows a similar trend, decreasing from 154.44 N at Khalal to 23.27 N at Rutab and increasing to 25.38 N at Tamar. Deformation at bioyield and rupture also changes across the stages, with a marked decrease at Rutab followed by a slight increase at Tamar. These results indicate that while the Saqie variety softens significantly at the Rutab stage, it partially regains firmness at the Tamar stage due to moisture loss and the concentration of fibrous material, with statistical significance (*p* < 0.05) noted in these observations. Also, for Khodry variety displays a similar pattern, with significant decreases in both the force at bioyield and rupture from Khalal to Rutab stages, followed by an increase at the Tamar stage. At Khalal, the force at bioyield is 79.90 N, which drops to 4.60 N at Rutab and then increases to 18.71 N at Tamar. The force at rupture shows a comparable trend, decreasing from 94.50 N at Khalal to 12.00 N at Rutab, and then increasing to 28.40 N at Tamar. The deformation at bioyield and rupture also reflects these changes, with a significant reduction at Rutab and an increase at Tamar. These changes are statistically significant (*p* < 0.05) and highlight the impact of ripening on the mechanical characteristics of Khodry dates, where the initial softening at Rutab is followed by a relative increase in firmness at Tamar due to the drying process and increased structural integrity.

The analysis of the force-deformation characteristics of Barhi, Saqie, and Khodry dates at different ripening stages reveals significant textural changes driven by variations in moisture content and cell structure integrity. The Barhi variety shows the most pronounced softening from Khalal to Rutab stages, while Saqie and Khodry varieties exhibit a similar pattern with an initial softening followed by a slight increase in firmness at the Tamar stage.

#### Date palm toughness

**Fig. 5:**
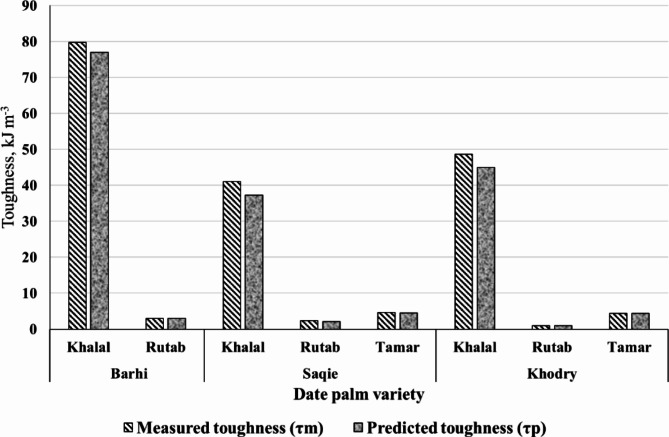
Measured toughness (*τm*) and predicted toughness (*τp*).

The results of measured toughness (*τm*) and predicted toughness (*τp*) are presented in Fig. 5. It’s showed that *τm* closely aligns with *τp*. For all varieties, the predicted toughness is approximately 0.0 to 12.4% lower than the measured toughness.

Significant textural differences are observed in the Barhi, Saqie, and Khodry dates at different ripening stages. The Barhi variety exhibits a sharp decline in toughness from the Khalal to Rutab stages, highlighting the impact of ripening on the fruit’s softening process. The Saqie variety shows an initial softening from the Khalal to Rutab stages, followed by a slight increase in toughness at the Tamar stage. Similar patterns are observed in the Khodry variety, where the fruit softens significantly from the Khalal to Rutab stages and then experiences a noticeable increase in toughness at the Tamar stage due to dryness and fiber concentration. The toughness of Barhi dates at the Khalal and Rutab stages shows a marked decrease as the fruit ripens, with measured toughness (*τm*) dropping significantly from 79.72 kJ • m^–3^ at the Khalal stage to 3.00 kJ • m^–3^ at the Rutab stage, indicating substantial softening. This decline is mirrored in the predicted toughness (*τp*), which decreases from 77.00 kJ • m^–3^ at Khalal to 3.00 kJ • m^–3^ at Rutab, with the percentage change between measured and predicted toughness reducing from 3.41% at Khalal. For the Saqie variety, toughness across the Khalal, Rutab, and Tamar stages illustrates significant variations during ripening. The measured toughness (*τm*) decreases from 41.00 kJ • m^–3^ at Khalal to 2.30 kJ • m^–3^ at Rutab, and then slightly increases to 4.60 kJ • m^–3^ at Tamar. Similarly, the predicted toughness (*τp*) follows this pattern, decreasing from 37.21 kJ • m^–3^ at Khalal to 2.14 kJ • m^–3^ at Rutab, and increasing to 4.50 kJ • m^–3^ at Tamar. The percentage change between measured and predicted toughness is highest at Khalal (9.24%). These statistically significant changes (*p* < 0.05) suggest that the Saqie variety undergoes considerable softening initially but slightly regains firmness at the Tamar stage due to dehydration and concentration of the fruit’s structural components, as supported by the findings of Hussain et al.^22^. The Khodry variety exhibits substantial changes in toughness characteristics from Khalal to Tamar stages. The measured toughness (*τm*) drops from 48.66 kJ • m^–3^ at Khalal to 1.00 kJ • m^–3^ at Rutab, and then increases to 4.40 kJ • m^–3^ at Tamar. The predicted toughness (*τp*) follows a similar trend, decreasing from 45.00 kJ • m^–3^ at Khalal to 1.00 kJ • m^–3^ at Rutab, and then increasing to 4.32 kJ • m^–3^ at Tamar, with the percentage change highest at the Khalal stage (7.52%). These findings are in line with Hamad et al.^24^, who noted that the dehydration and concentration of sugars in dates during the Tamar stage contribute to the slight increase in toughness observed.

This study of date toughness at various ripening stages has practical significance for farmers regarding storage and marketability. The pronounced decrease in toughness from the Khalal to Rutab stages, particularly in the Barhi variety, indicates an increased susceptibility to deformation and spoilage during storage as dates ripen. Understanding these textural changes enables farmers to optimize harvest timing based on their storage needs and target markets. For example, firmer dates at the Khalal stage may be ideal for long-term storage and export, whereas dates at the Rutab stage require careful handling and shorter storage times due to their reduced structural integrity. Additionally, the slight increase in toughness observed at the Tamar stage, especially for Saqie and Khodry varieties, suggests that further drying could help maintain firmness for longer storage periods, enhancing fruit quality and longevity.

## Conclusion

This study aimed to predict the toughness of date palm fruit (Barhi, Saqie, and Khodry varieties) across ripening stages (Khalal, Rutab, and Tamar) using Hertz Theory by analyzing physical and mechanical characteristics. Results showed significant decreases in measured and predicted toughness from Khalal to Rutab due to ripening-induced softening. For Barhi, measured toughness (*τm*) dropped from 79.72 kJ • m^–3^ at Khalal to 3.00 kJ • m^–3^ at Rutab, closely matching predicted toughness (*τp*) values, with only a slight variation (~ 3.41%). Mechanical characteristics, including elasticity modulus and rupture stress, also decreased as dates ripened, reflecting reduced firmness and resistance. Saqie and Khodry varieties exhibited similar trends, with noticeable toughness reductions from Khalal to Rutab, followed by a slight increase at Tamar due to moisture loss and fiber concentration. For instance, Saqie’s measured toughness decreased from 41.00 kJ • m^–3^ at Khalal to 2.30 kJ • m^–3^ at Rutab, then slightly increased to 4.60 kJ • m^–3^ at Tamar, aligning closely with predicted values and supporting Hertz Theory’s accuracy in predicting these characteristics. Mechanical characteristics, like bioyield stress and rupture stress, showed statistically significant reductions (*p* < 0.05) across all varieties, indicating the fruits’ progression toward softness and deformation as they matured. These findings underscore the impact of ripening on date texture, particularly from Khalal to Rutab stages, where rapid softening occurs. The Tamar stage, with its slightly higher toughness, appears more suitable for storage and processing. The high agreement between predicted and measured toughness across varieties suggests that Hertz Theory effectively models date fruit toughness, providing a valuable tool for optimizing harvesting and storage strategies.

Further study: Optimizing Storage and Handling Techniques for Date Palm Varieties by Assessing Ripening Stages and Drying Effects on Fruit Toughness and Shelf Life.

## Data Availability

The datasets generated and/or analyzed of the current study are available from the corresponding author on reasonable request.

## Plant guideline statement

The date palm fruit varieties (Barhi, Saqie, and Khodry) were gifted from a private farm (Sheikh Mohammed Abdul Aziz Al-Rajhi), Qassim region, Saudi Arabia.
